# Expression of Concern: Uterine leiomyoma is associated with the risk of developing endometriosis: A nationwide cohort study involving 156,195 women

**DOI:** 10.1371/journal.pone.0339078

**Published:** 2025-12-18

**Authors:** The PLOS One Editors

The *PLOS One* Editors issue this Expression of Concern because this article [1] was identified as one of a series of submissions for which we have concerns about the peer review process. Readers are advised to interpret the article [1] with caution.

The Editors have no evidence of any author involvement in the peer review concerns.

## References

[1] Lin KY-H, Yang C-Y, Lam A, Chang CY-Y, Lin W-C. Uterine leiomyoma is associated with the risk of developing endometriosis: A nationwide cohort study involving 156,195 women. PLoS One. 2021;16(8):e0256772. doi: 10.1371/journal.pone.0256772 34437644 PMC8389431

